# SPEMix: a lightweight method via superclass pseudo-label and efficient mixup for echocardiogram view classification

**DOI:** 10.3389/frai.2024.1467218

**Published:** 2025-01-08

**Authors:** Shizhou Ma, Yifeng Zhang, Delong Li, Yixin Sun, Zhaowen Qiu, Lei Wei, Suyu Dong

**Affiliations:** ^1^College of Aulin, Northeast Forestry University, Harbin, China; ^2^College of Computer and Control Engineering, Northeast Forestry University, Harbin, China; ^3^The Department of Ultrasound, Harbin Medical University Cancer Hospital, Harbin, China; ^4^Department of Cardiovascular Surgery, First Affiliated Hospital With Nanjing Medical University, Nanjing, China

**Keywords:** superclass pseudo-label, lightweight, semi-supervised, open-set, echocardiogram view classification

## Abstract

**Introduction:**

In clinical, the echocardiogram is the most widely used for diagnosing heart diseases. Different heart diseases are diagnosed based on different views of the echocardiogram images, so efficient echocardiogram view classification can help cardiologists diagnose heart disease rapidly. Echocardiogram view classification is mainly divided into supervised and semi-supervised methods. The supervised echocardiogram view classification methods have worse generalization performance due to the difficulty of labeling echocardiographic images, while the semi-supervised echocardiogram view classification can achieve acceptable results via a little labeled data. However, the current semi-supervised echocardiogram view classification faces challenges of declining accuracy due to out-of-distribution data and is constrained by complex model structures in clinical application.

**Methods:**

To deal with the above challenges, we proposed a novel open-set semi-supervised method for echocardiogram view classification, SPEMix, which can improve performance and generalization by leveraging out-of-distribution unlabeled data. Our SPEMix consists of two core blocks, DAMix Block and SP Block. DAMix Block can generate a mixed mask that focuses on the valuable regions of echocardiograms at the pixel level to generate high-quality augmented echocardiograms for unlabeled data, improving classification accuracy. SP Block can generate a superclass pseudo-label of unlabeled data from the perspective of the superclass probability distribution, improving the classification generalization by leveraging the superclass pseudolabel.

**Results:**

We also evaluate the generalization of our method on the Unity dataset and the CAMUS dataset. The lightweight model trained with SPEMix can achieve the best classification performance on the publicly available TMED2 dataset.

**Discussion:**

For the first time, we applied the lightweight model to the echocardiogram view classification, which can solve the limits of the clinical application due to the complex model architecture and help cardiologists diagnose heart diseases more efficiently.

## 1 Introduction

**Figure 1:**
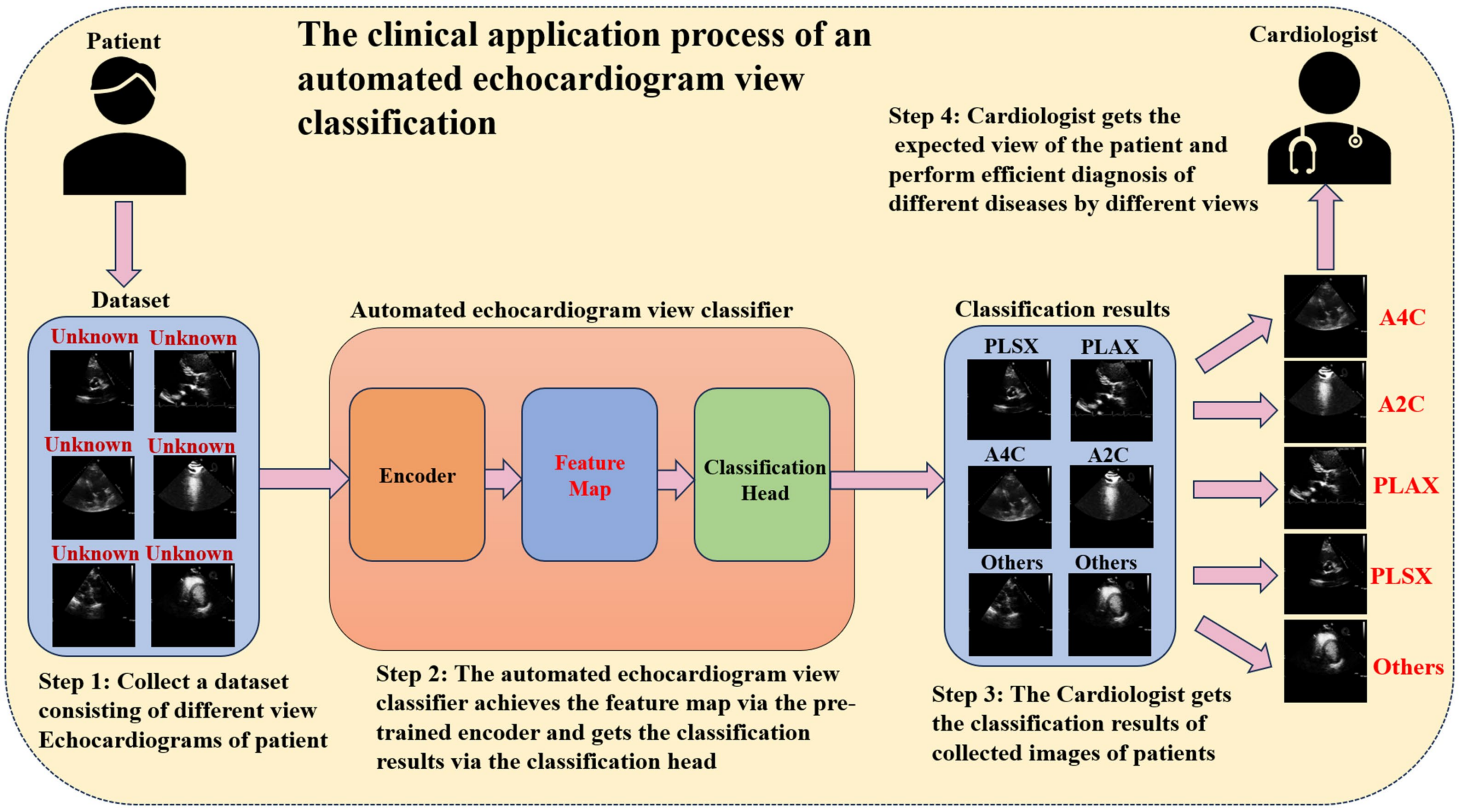
The clinical application process of an automated echocardiogram view classification.

When diagnosing heart diseases based on the echocardiogram, different heart diseases depend on different views of the echocardiogram. For example, aortic stenosis can be diagnosed by analyzing the PLAX and PSAX views (Huang et al., 2021), and early myocardial infarction can be detected via A4C and A2C (Degerli et al., 2024). Hence, cardiologists usually need to identify the critical echocardiogram view during the clinical diagnostic process. Automated echocardiogram view classification can effectively reduce the clinical diagnosis time (Zhu et al., 2022). Figure 1 illustrates the clinical application process of the automated echocardiogram view classification. In practical clinical applications, automated view classification involved four steps. First, the cardiologist collected the echocardiogram data from the patient. Then, the cardiologist inputted the data into the automated view classification model to perform the echocardiogram view classification. Next, the cardiologist got the classification results of the echocardiogram. Finally, the cardiologist selected the expected view when making a diagnosis of a different disease and performed the diagnosis. Obviously, the core of automated echocardiogram view classification methods is to classify by the classification model. In this situation, developing a classification model that can identify the critical view precisely and efficiently will be vital in medicine. So many studies aim to develop echocardiogram view classification methods to assist cardiologists in diagnosing heart disease.

With the development of deep learning in medical images, there are many studies (Kusunose et al., 2020; Madani et al., 2018; Gao et al., 2017) focused on the classification of echocardiogram views by deep learning. These methods can be divided into supervised methods and semi-supervised methods.

Supervised studies used a large number of labeled data to train a model. Madani et al. (2018) performed the classification task by building a convolution model consisting of traditional convolution layers and fully connected layers. They classified 15 different echocardiogram views and got impressive classification results on their private dataset. Kusunose et al. (2020) collected 17,000 echocardiogram images from 340 patients and built a neural network model. The model consisted of five convolution layers and five pooling layers to classify them into five categories. Gao et al. (2017) fused the spatial and temporal information to perform eight view classifications based on their 432 video data. Although these supervised methods have gotten good results in identifying or detecting tasks (Gao et al., 2017; Han et al., 2024; Han et al., 2024), they need massive labeled data. Unfortunately, it will take a lot of time and energy for medical experts to label the medical images. Furthermore, these studies (Kusunose et al., 2020; Madani et al., 2018; Gao et al., 2017) used proprietary datasets, which made the generalization of their methods cannot be guaranteed. The private datasets also made it challenging to apply actual clinical needs. To address these issues, our study focused on the development of a semi-supervised algorithm within the publicly available dataset. We evaluated the generalization of our method in two different public datasets to prove the feasibility of our SPEMix in clinical application.

Semi-supervised studies (Madani et al., 2018; Hagberg et al., 2022) tried to decrease the cost of labeling data and improve efficiency by utilizing small labeled data and massive unlabeled data. Therefore, semi-supervised learning has been revealed to have superior potential (Xiaojin, 2008), and it has also been widely used in medicine (Chebli et al., 2018; Bai et al., 2017). Many scholars (Madani et al., 2018; Hagberg et al., 2022; Huang et al., 2023; Huang et al., 2024) have used semi-supervised learning for the echocardiogram view classification task. Madani et al. (2018) developed a semi-supervised generative adversarial network model to classify 15 views of echocardiogram via only a little labeled data. This method revealed the huge potential of semi-supervised. Hagberg et al. (2022) innovatively developed a semi-supervised learning method using natural language processing for right ventricle view classification, which improved the efficiency of classification. Although these methods had good classification efficiency, their classification accuracy was usually worse than the classification accuracy of supervised methods due to ignoring the damage caused by the out-of-distribution data. Most current methods have not achieved accurate and reliable classification accuracy to meet practical clinical needs. We proposed a novel efficient mixup method for our semi-supervised method to solve the above problem. Different from other mixing methods (Zhang et al., 2018; Yun et al., 2019; Liu et al., 2022) applied to natural images, our proposed efficient mixup method can automatically focus on the valuable pixel of the echocardiogram based on dynamic attention and produce high-quality augmented echocardiogram images. Specifically, we proposed a DAMix Block for our SPEMix to achieve our efficient augmentation.

Otherwise, many studies have observed that out-of-distribution of unlabeled data can harm the accuracy of semi-supervised learning (Oliver et al., 2018; Li et al., 2023; Zhao et al., 2022). Out-of-distribution data means the data does not belong to any of the known classification categories. The semi-supervised methods achieved good results only when the unlabeled data shared the same class space with labeled data. Unfortunately, the collected unlabeled medical image datasets often included out-of-distribution data in practical medical applications. This phenomenon usually leads to unlabeled data sharing a different class space with labeled data. Some approaches tried to improve the medical image classification accuracy via adversarial attacks and defenses (Lee et al., 2024; Kwon, 2023; Kwon and Lee, 2023). However, the dominant approaches to tackle the harm caused by the out-of-distribution data in natural images were to detect and filter the out-of-distribution data (Saito et al., 2021; Calderon-Ramirez et al., 2022). Huang et al. (2023) used augmentation and step direction modification to reduce the harm of out-of-distribution for echocardiogram view classification. The proposed method utilized the gradient information from unlabeled data from out-of-distribution only when the out-of-distribution data could improve the model performance. Different from the previous techniques, filtering out the out-of-distribution or modifying the gradient descent updates, we designed a novel, effective open-set framework to make better use of the out-of-distribution unlabeled data. Inspired by the success of the superclass work (Li et al., 2023) in solving the harm of out-of-distribution, we modeled the unlabeled data from superclass distribution. We assigned the superclass pseudo-label for unlabeled data. Specifically, we regarded all of the classes out of the distribution as a new superclass, and we designed the SP Block to generate superclass pseudo-labels to leverage the information in unlabeled datasets better. The SP Block included a multiclass classifier and a close-set classifier to calculate the out-of-distribution class probability distribution and the in-the-distribution class probability distribution. SP Block can calculate the superclass probability distribution and generate the superclass pseudo-labels based on these probabilities. Then, our superclass pseudo-labels can be leveraged by an open-set classifier, and our model can learn the semantic features of unlabeled data that are out of the distribution.

On the other hand, some researcher (Avola et al., 2024) focused on developing more complex models to improve the accuracy of classification. Avola et al. (2024) built a multi-scale feature transformer model to capture different scale details to get better classification results in the TMED2 (Huang et al., 2022) dataset. Although this model can provide multi-view and single-view recognition, the transformer model has a more significant number of parameters and lower computational efficiency compared with the lightweight models. To satisfy real medical clinical needs, for the first time, we applied the lightweight model to echocardiogram view classification to improve the classification efficiency. RepViT (Wang et al., 2024) model designed a novel lightweight CNN based on the structures of lightweight VIT and achieved the best performance of the lightweight model. Inspired by the great work, we designed a four-layer lightweight encoder based on the RepViT for view classification. To improve our training efficiency, we decoupled the mixed data generate stage and the pseudo-label generate stage. Specifically, we embedded the DAMix Block into the teacher encoder to provide high-quality mixed data to the classifier. We embedded the SP Block into the student model to leverage the out-of-distribution.

In this paper, we proposed a lightweight open-set semi-supervised method, SPEMix, for echocardiogram view classification. Different from traditional semi-supervised learning classification algorithms, such as FixMatch and OpenMatch, our proposed SPEMix can classify the echocardiogram views accurately and efficiently, providing a new perspective on practical clinical application. FixMatch achieves semi-supervised learning by assigning pseudo-labels to unlabeled data but discards unlabeled data with low thresholds, making it difficult to consider unseen classes. In contrast, our SPEMix assigns open-set pseudo-labels to each unlabeled data point, leveraging the semantic information of each unlabeled instance. Compared to OpenMatch, our open-set semi-supervised method introduces mixup data augmentation, which improves classification accuracy through joint supervision of unseen classes and mixed data. In summary, our contributions are as follows:

1. We innovatively proposed a mixed data generator (DAMix) which introduced the dynamic attention mechanism for the medical echocardiogram. The DAMix Block can provide the mixed mask embedded with the mixing ratio. The mixed mask, which consists of valuable information, can mix up the unlabeled echocardiogram efficiently at the pixel level.
2. We proposed a novel method to leverage the out-of-distribution unlabeled data from superclass probability contribution. We designed an SP Block to model the unlabeled data in terms of superclass distribution to generate the pseudo-labels of unlabeled data. Then, we introduced an open-set classifier to calculate the superclass prediction. We also proposed a novel open-set loss based on consistent regularization to utilize the superclass pseudo-label.
3. Finally, we applied the lightweight model to our semi-supervised method and built a lightweight encoder based on RepViT, which improved the accuracy and efficiency of our proposed method.

## 2 Methods

**Figure 2:**
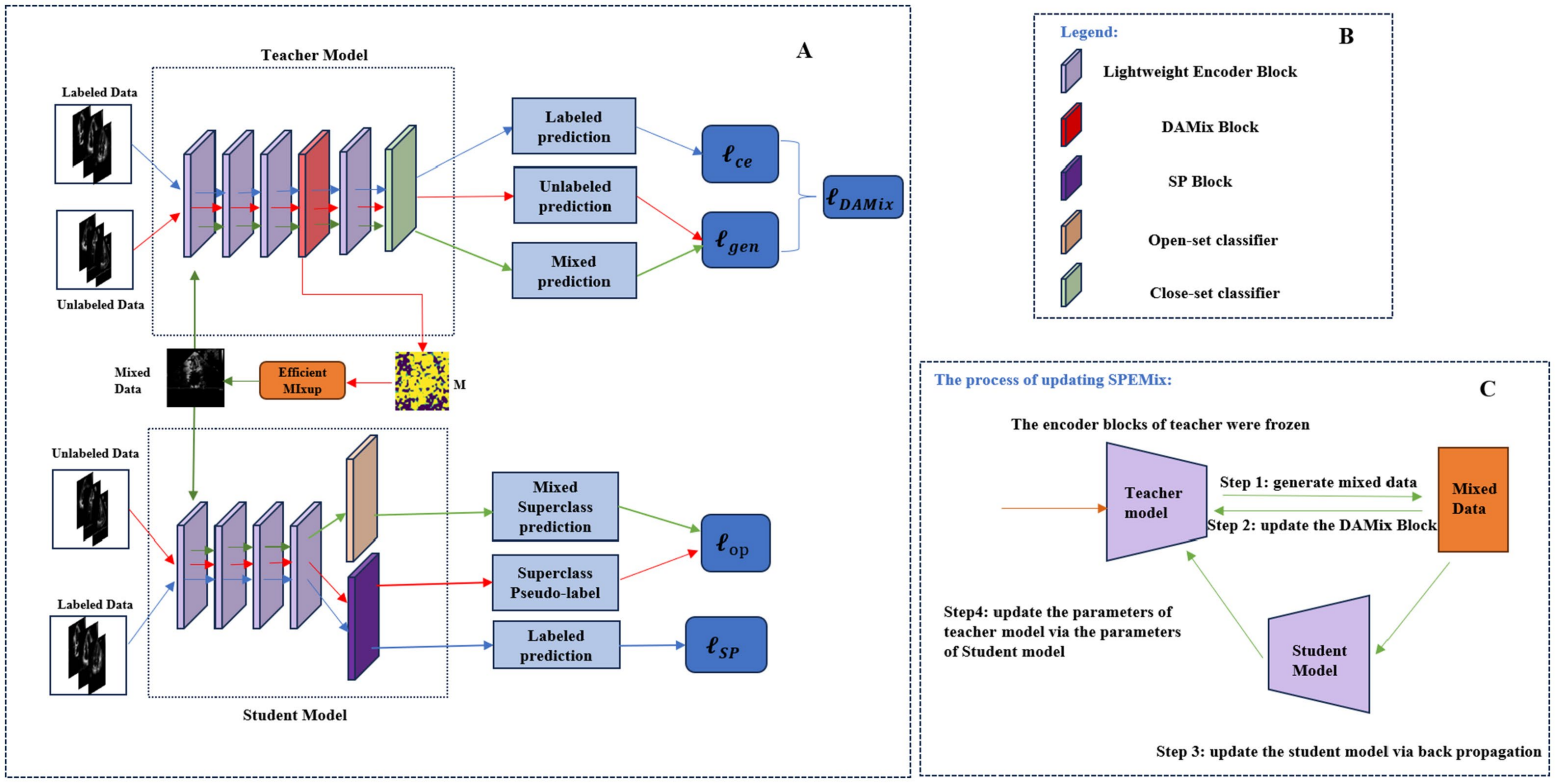
The framework of the proposed method **(A)**. Specifically, the DAMix Block was embedded behind the third layer of the teacher model and generated the mixup mask to mix up the input of the unlabeled data. The SP Block was added at the end of the student model to generate the superclass pseudo-label. The open-set classifier can calculate the superclass prediction. The legend of the framework **(A)** is shown in **(B)**. The process of updating SPEMix is shown in **(C)**. The parameters of the student model were updated via backpropagation, and the teacher parameters were updated by the exponential moving average strategy of the student model parameters.

In this section, we introduced the detailed information of the proposed novel framework, SPEMix, for echocardiogram view classification. The framework of the proposed SPEMix was shown in Figure 2. The two core components of the SPEMix, DAMix Block and SP Block, can, respectively, generate the mixed mask to mix up data and superclass pseudo-label to leverage the out-of-distribution data. Meanwhile, the SPEMix used the lightweight encoder model to improve efficiency. The lightweight teacher model consisted of the DAMix Block to generate the augmentation data. The lightweight student model consisted of the SP Block to generate the superclass pseudo-label. The teacher model and student model were trained in an end-to-end manner to enable data enhancement and superclass pseudo-labels generation to work together.

### 2.1 DAMix block for the generation of high-quality augmented echocardiograms

**Figure 3:**
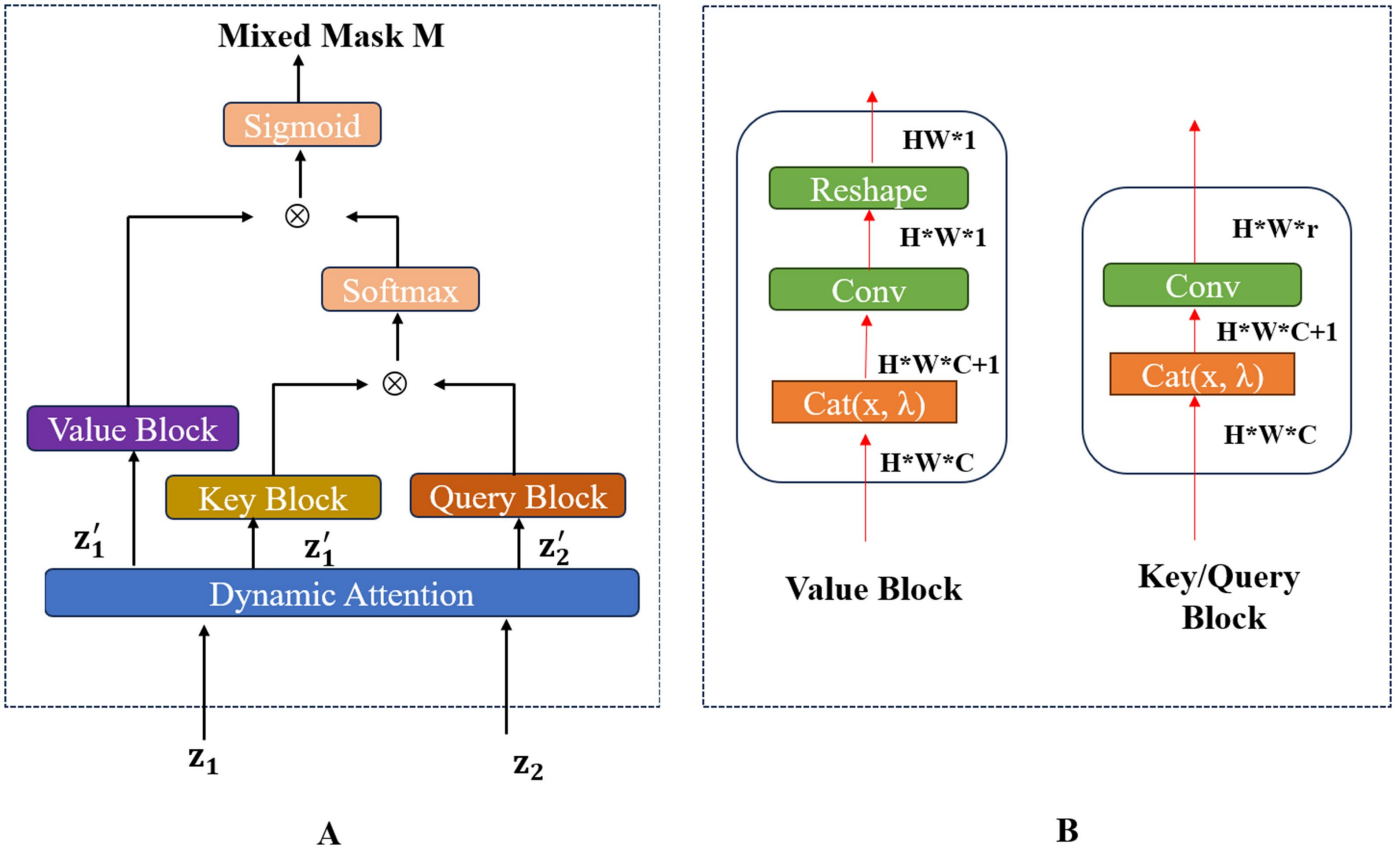
The overall framework of the DAMix Block is shown in **(A)**. The structure of the Value Block, Key Block and Query Block is shown in **(B)**.

Mixing augmentation technology (Zhang et al., 2018) is useful for improving the accuracy and generalization of classification tasks. However, the traditional mixing augmentation methods did not work well for echocardiogram view classification because most echocardiograms are grayscale images (Shorten and Khoshgoftaar, 2019). To address these issues and improve the accuracy and generalization of echocardiogram view classification, we proposed DAMix Block to perform pixel-level Efficient Mixup. The DAMix Block generated the mixed mask M that contains crucial information for performing the pixel-level Efficient Mixup process. This DAMix Block was capable of embedding the mixing ratio into the mixed mask. The framework of the DAMix Block was shown in Figure 3.

Inspired by the improvement of the sparse self-attention in the vision transformer (Zhu et al., 2023), the DAMix Block first introduced the dynamic attention mechanism for the echocardiogram. The dynamic attention mechanism divided the echocardiogram into sub-regions and searched for the regions containing critical information in the feature map. This process helped the generated mask to capture the global features of the entire image efficiently. The process of the dynamic attention mechanism was as follows: Initially, the dynamic attention mechanism divided the echocardiogram feature $x\in {\mathbb{R}}^{H\times W\times C}$ into $S^{2}$ different small square regions, so the size of each region will be $\frac{HW}{S^{2}}$ . We regarded each of the square regions as a token. Each token had $\frac{HW}{S^{2}}$ features and there were $S^{2}$ tokens in total. This process can be achieved by reshaping the feature map into $x^{\prime }\in {\mathbb{R}}^{\frac{HW}{S^{2}}\times S^{2}\times C}\;\text{.}$ After the divided step, we will get the echocardiogram token features $x^{\prime }$ . Then, the dynamic attention mechanism calculated the region Query(Q), region Key(K), and region Value(V) of the token features $x^{\prime }$ by linear projections. The process is shown in Equation 1:


(1) $$Q=x^{\prime }W^{q},K=x^{\prime }W^{k},V=x^{\prime }W^{v}$$

Where $W^{q}$ was the linear projection weight of the region query, $W^{k}$ was the linear projection weight of the region key, and $W^{v}$ was the linear projection weight of the region value.

Furthermore, the dynamic attention mechanism calculated the average matrix of the region Query Q, and region Key K . Specifically, this dynamic attention can calculate the average feature of each token. This process can be achieved by performing 2D average pooling with a kernel size of $\frac{HW}{S^{2}}\times 1$ and reshaping the pooling results into $S^{2}\times$ C as the average matrix $Q^{a}\in {\mathbb{R}}^{S^{2}\times C}$ and $K^{a}\in R^{S^{2}\times C}$ .

To search for the crucial regions of the echocardiogram efficiently, the similarity between each region was calculated by the result of the dot product of the average matrix of the query and key. The process of calculating the similarity is shown in Equation 2:


(2) $$P=Q^{a}{\left(K^{a}\right)}^{T}$$

Where $P\in {\mathbb{R}}^{S^{2}\times S^{2}}$ denoted the similarity of the regions; $Q^{a}\in {\mathbb{R}}^{S^{2}\times C}$ denoted the average matrix of the Q, $K^{a}\in R^{S^{2}\times C}$ denoted the average matrix of the K . The last step of the dynamic-attention mechanism was to find the index $\left(id_{1},id_{2},id_{3},\ldots ,id_{k}\right)$ of the top k most relevant tokens for each token, then we collected all of the relevant tokens of K and V as a new $K^{c}$ and a new $V^{c}$ , which can be achieved by searching for the index of P . The dynamic attention can be represented as the Equation 3:


(3) $$\mathrm{output}=\mathrm{softmax}\left(\frac{Q{\left(K^{c}\right)}^{T}}{\sqrt{c}}\right)V^{c}$$

DAMix Block was guided by the dynamic attention mechanism to find regions in the echocardiogram that are useful for the classification task. In addition, DAMix Block embedded the mixing rates into the corresponding feature maps and used the idea of cross-attention to generate appropriate mixed masks for the echocardiogram. The process of mixed mask generation can be formulated as follows: getting an unlabeled image pair $\left(u_{1},u_{2}\right)$ from the minibatch of the unlabeled dataset. The feature maps from the k-th layer of the unlabeled image pair were inputted into the DAMix Block. The dynamic attention module was subsequently employed to calculate the weighted feature maps $z_{1}^{\prime },z_{2}^{\prime }$ . Furthermore, to achieve the pixel-level mixup, we designed the Value Block, Query Block, and Key Block to calculate the Value matrix embedded with *λ*, Query matrix embedded with λ, and Key matrix embedded with λ respectively, where λ $\in \left[0,1\right]$ is the mixing ratio that satisfies Beta distribution $\lambda -\beta \left(\alpha ,\alpha \right)$ . Specifically, these modules can splice a mixing rate matrix of the same size as the feature over the channel dimension of the feature, where each element of the mixing rate matrix was a randomly generated lambda λ. Then the DAMix Block got the Query matrix Q, Value matrix V, and Key matrix K, respectively, via the query block, value block, and key block. The structure of these blocks were shown in the right part of Figure 3. Notably, the Key block had the same structure as the Query block. To get the mixed mask M , we calculated the similarity matrix P of Q and K by the cross-attention mechanism, and generated the mixed mask M by using the similarity matrix $P_{2}$ and Value matrix V. The function of generating M can be formulated as the Equations 4^5^:


(4) $$P_{2}=\mathrm{softmax}\left(\frac{K{\left(z_{1}^{\prime }\right)}^{T}⊗Q\left(z_{2}^{\prime }\right)}{C}\right)$$

(5) $$M=U\left(\sigma \left(P_{2}⊗V\left(z_{1}^{\prime }\right)\right)\right)$$

Where σ was the Sigmoid activation function, V denoted the value block, K denoted the key block, Q denoted the query block, ⊗ denoted the matrix multiplication, and C was a normalization factor, U denoted upsample function.

Based on the mixed masks generated by DAMix Block, we proposed the Efficient Mixup. The Efficient Mixup will generate high-quality mixed images via the mixed mask. Efficient Mixup achieved linear interpolation through pixel-wise multiplication between data and masks, generating high-quality mixed echocardiograms. When performing the efficient mixup between $u_{1}$ and $u_{2}$ , we regard unlabeled image $u_{1}$ as the value. The DAMix Block calculated the mixed mask M for the value image. Similarly, the mixed mask of the value image $u_{2}$ was 1-M generated via the DAMix Block. The specific formula for efficient mixup was the Equation 6:


(6) $$\mathrm{Efficient Mixup}=M⊙u_{1}+\left(1-M\right)⊙u_{2}$$

**Figure 4:**
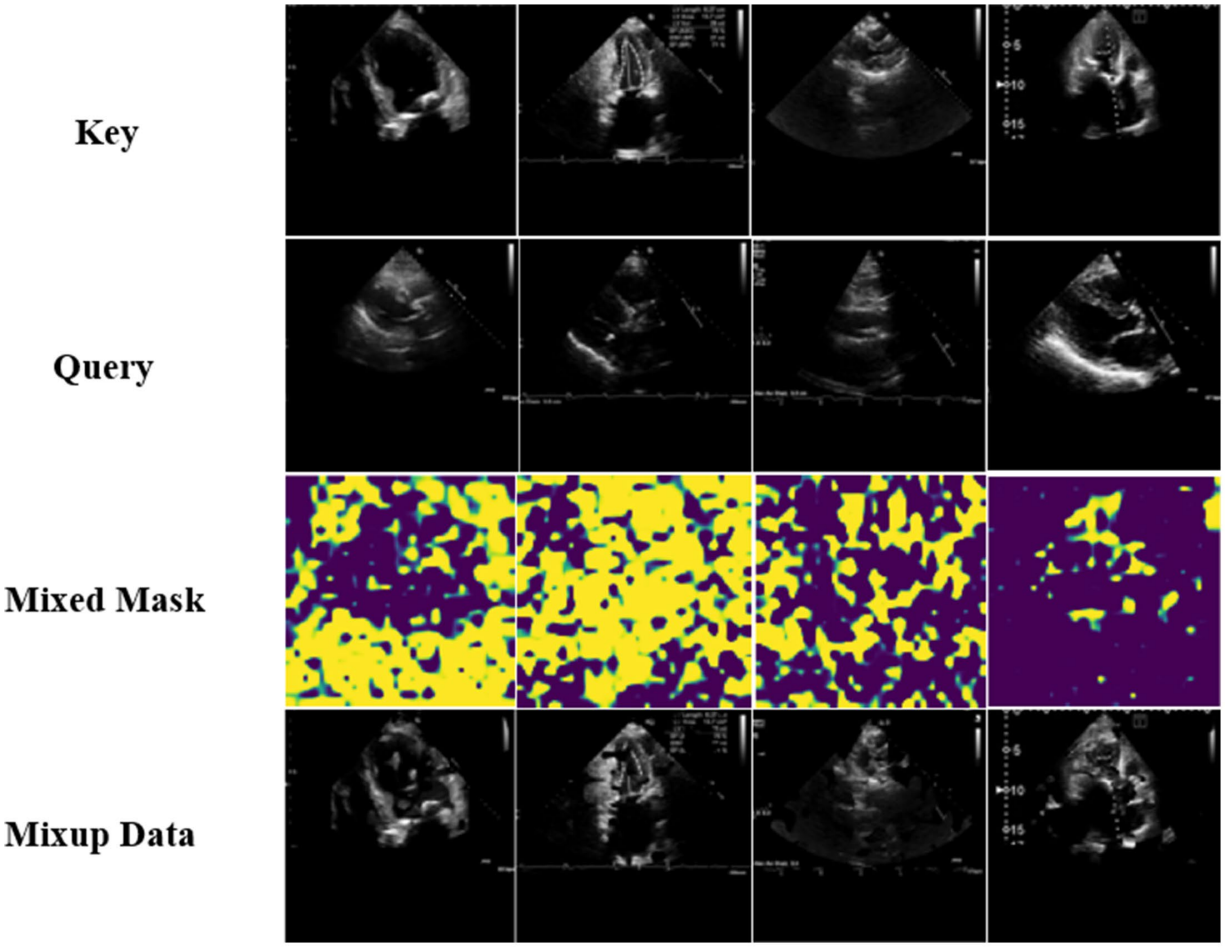
Visualization results of the mixed masks generated via DAMix Block and visualization results of mixup data generated via Efficient Mixup.

Where ⊙ denoted element-wise product, $u_{\mathrm{mix}}$ was the output result of the DAMix Block. The visualization results of the mixed masks generated via DAMix Block and visualization results of mixup data generated via Efficient Mixup were shown in the Figure 4.

In the training process of our SPEMix, following the previous successful work (Liu et al., 2022), we also embedded our DAMix Block behind the third layer of the encoder. The DAMix loss function can update the parameters of the DAMix Block, a combination of the unlabeled augmentation data generation loss and the labeled data classification loss. To calculate the augmentation generation loss, we proposed the novel generation loss based on the mixed cross-entropy loss function (Liu et al., 2022) for the mixed unlabeled augmentation data. In the previous work, the cross entropy loss between mixed prediction and the mixup of labels was represented by the mixed cross-entropy loss function. Hence, we calculate our mixed generation loss by modifying the mixed cross-entropy. The view of our unlabeled mixed cross-entropy loss function was as follows: we considered the prediction $p_{1},p_{2}$ of unlabeled data $u_{1},u_{2}$ before augmentation as the label of the unlabeled data. The generation loss function can be formulated as the Equation 7:


(7) $${𝓁}_{gen}=\lambda {𝓁}_{ce}\left(p_{1},p_{\mathrm{mix}}\right)+\left(1-\lambda \right){𝓁}_{ce}\left(p_{2},p_{\mathrm{mix}}\right)$$

Where λ meant the mixing ratio, and ${𝓁}_{ce}$ meant the cross-entropy loss function.

However, the encoder’s predictions of the unlabeled data before augmentation were unreliable in the early stages of training, which leaded to the wrong optimization of the generation loss. To address this issue, we also used the supervision of labeled data to assist in the generation of the mixup mask to guide the process of optimizing the DAMix Block. The process can be achieved by the cross-entropy of the labeled data, the process can be formulated as the Equation 8:


(8) $${𝓁}_{\mathrm{labeled}}={𝓁}_{ce}\left(y,p_{x}\right)$$

where y denoted the ground truth of the labeled data, and $p_{x}$ denoted the prediction of the labeled data. Finally, the loss of the DAMix Block denoted the combination of the generation loss and the cross-entropy loss of the labeled data, be formulated as the Equation 9:

(9) $${𝓁}_{\mathrm{DAMix}}={𝓁}_{gen}+{𝓁}_{\mathrm{labeled}}$$

### 2.2 SP block for the generation of the echocardiogram superclass pseudo-label

To utilize the unlabeled data of out-of-distribution, we proposed a novel superclass pseudo-label from the perspective of superclass probability. Specifically, our approach first considered all in-the-distribution classes as the in-the-distribution superclass and all out-of-distribution class data as an out-of-distribution superclass. Then, we modeled the superclass from the perspective of the probability distribution to generate the superclass pseudo-label.

**Figure 5:**
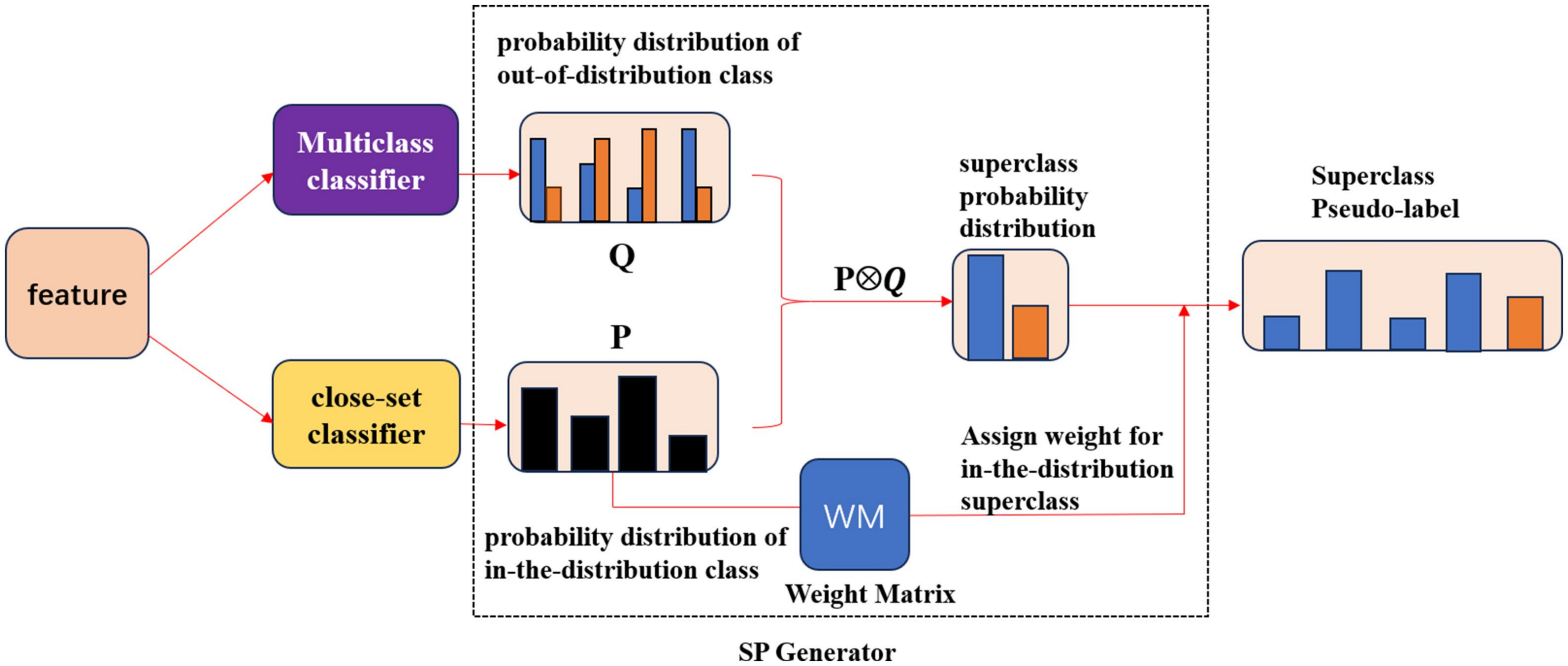
The framework of the SP Block. Specifically, the multiclass classifier calculated the probability distribution of out-of-distribution class Q, $Q\in {\mathbb{R}}^{4\times 2}\text{.}$ The close-set classifier calculated the probability distribution of in-the-distribution class P, $P\in {\mathbb{R}}^{1\times 4}\text{.}$ Then the SP Generator acquired the superclass probability distribution and generated the Superclass Pseudo-label by assigning the weight matrix for the in-the-distribution superclass.

We designed the SP Block(short for Superclass Pseudo-label generator) to achieve the above process. The framework of the SP Block is shown in Figure 5. The SP Block included a multiclass classifier to calculate the out-of-distribution class probability distribution, a close-set classifier to calculate the in-the-distribution class probability distribution, and a SP Generator to get the superclass pseudo-label. The black column of P in Figure 5 represented the probability of the sample belonging to each in-the-distribution class only considering the presence of the in-the-distribution classes. The blue columns of Q in Figure 5 represented the probability of the sample belonging to each in-the-distribution class accounting for the unknown classes. Notably, the black column of P was not the same as the blue column of Q. While the orange columns of Q in Figure 5 represented the probability of the sample not belonging to each in-the-distribution class.

Our SP Block generated the superclass pseudo-label for unlabeled samples from the perspective of superclass probability distributions. Our SP Block acquired the in-the-distribution probability distribution P by calculating the probability that the sample belongs to each class in the distribution. Specifically, the close-set classifier, which is a normal fully connected layer, calculated the in-the-distribution probability P, P∊ ${\mathbb{R}}^{1\times 4}$ . However, we can only get the true class prediction when this unlabeled sample belongs to the in-the-distribution classes. Furthermore, we calculated the out-of-distribution probability distribution Q to model the out-of-distribution classes, which can be calculated by multi-binary classifiers. The multi-binary classifier has been proven to be able to detect whether a sample belongs to each in-the-distribution class,and the technology has been used widely in the previous open semi-supervised learning of filtering the out-of-distribution (Saito et al., 2021). The multiclass classifier consisted of four binary classifiers and each binary classifier predicted the sample whether belonging to the k-th class. Specifically, $\Psi_{k}$ is a multiclass classifier consisted of k binary classifier $\Psi_{k}=$ { $\varphi_{1},\varphi_{2},\varphi_{3},\ldots \varphi_{k}\}$ . $\varphi_{k}$ can output $o=\left(q_{k},1-q_{k}\right)\text{,}$ where $q_{k}$ means the probability belongs to the k-th class of the sample. The output of $\Psi_{k}$ was a matrix of k rows and 2 columns.

Then the SP Generator combined Q and P to generate the superclass pseudo-label, which can be achieved by the matrix multiplication of P and Q. The process of generating the superclass pseudo-label by the SP Generator had three steps: First, the SP Generator calculated the superclass probability distribution $D_{p}=\left(D_{\mathrm{in}},D_{\mathrm{out}}\right)\in {\mathbb{R}}^{1\times 2}$ , where $D_{\mathrm{in}}$ denoted the in-the-distribution superclass probability, $D_{\mathrm{out}}$ denoted the out-of-distribution superclass probability. Second, the SP Generator got the in-the-class probability weight matrix WM of the sample. Finally, the SP Generator assigned weights for the in-the-distribution superclass according to WM and got the superclass pseudo-label. The process of generating a superclass pseudo-label can be formulated as the process of Equations 10–12:


(10) $$D_{p}=P⊗Q$$

(11) $$WM=\left(\alpha_{1},\alpha_{2},\ldots \alpha_{n}\right)=\left(\frac{p_{1}}{\sum_{i=1}^{n}p_{i}},\frac{p_{2}}{\sum_{i=1}^{n}p_{i}},\ldots ,\frac{p_{i}}{\sum_{i=1}^{n}p_{i}}\right)$$

(12) $$SP=\left(WMD_{\mathrm{in}},D_{\mathrm{out}}\right)$$

Where $WM\in R^{1\times n}$ , where n denoted the number of the in-the-distribution class. SP denoted the superclass pseudo-label. $p_{i}$ denoted the i-th element of the in-the-distribution probability P.

To optimize the SP Block, we, respectively, optimized the multiclass classifier and the close-set classifier with labeled data. For the loss of the multiclass classifier, we used the hard-negative sampling strategy (Saito and Saenko, 2021), following the previous work. The loss function of the multiclass classifier can be formulated as the Equation 13:


(13) $${𝓁}_{mul}=\frac{1}{B}\overset{B}{\underset{i=1}{\sum }}\left(-\log\left(p_{i,y_{i}}\right)-lk\neq y_{i}\min og\left(\bar{p_{k}}\right)\right)$$

Where B represented the batch size of the labeled data, $p_{i,y}$ represented the first element in the y-th row of the out-of-distribution probability Q. $\bar{p_{k}}$ represented the second element in the k-th row of the out-of-distribution probability Q.

For the loss of the close-set classifier, we used the cross-entropy loss function of the labeled data to optimize, was shown in the Equation 14:


(14) $${𝓁}_{\mathrm{close}}={𝓁}_{ce}\left(y,p_{x}\right)$$

(15) $${𝓁}_{SP}={𝓁}_{\mathrm{close}}+{𝓁}_{mul}$$

The total loss of the SP Block was shown in Equation 15. In our SPEMix, we hoped the proposed DAMix Block and SP Block could work together to utilize the unlabeled datasets efficiently. Specifically, the SP Block can assign the superclass pseudo-label for augmentation data generated by the DAMix Block. To achieve the process, we built an open-set classifier to predict the superclass pseudo-label. We proposed the open-set loss function of SP Block based on the mixed process of the superclass pseudo-label to utilize the augmentation of unlabeled images. Specifically, the processes of calculating the open-set loss function are shown in the Equations 16–18:


(16) $${𝓁}_{\mathrm{op}}=\lambda {𝓁}_{ce}\left(y_{spu1},o_{\mathrm{mix}}\right)+\left(1-\lambda \right){𝓁}_{ce}\left(y_{spu2},o_{\mathrm{mix}}\right)$$

(17) $$y_{spu1}=\max\left(SP_{u1}\right)$$

(18) $$y_{spu2}=\max\left(SP_{u2}\right)$$

Where $SP_{u1}$ meant the superclass pseudo-label of $u_{1}$ , $SP_{u2}$ meant the superclass pseudo-label of $u_{2}$ . $y_{spu2}$ meant the corresponding class of the pseudo-label $SP_{u2}$ . $y_{spu1}$ meant the corresponding class of the pseudo-label $SP_{u1}$ . max was the function to caculate the index of the maximum probability. $o_{\mathrm{mix}}$ denoted the superclass pseudo-label prediction of augmentation image from $u_{1}$ and $u_{2}$ via the open-set classifier.

### 2.3 Lightweight encoder and end-to-end efficient learning paradigm

For the first time, we applied the lightweight model to the echocardiogram view classification. Specifically, we designed a lightweight encoder based on the RepViT (Wang et al., 2024) network for our SPEMix. RepViT implemented the design of lightweight networks by using the structural re-parameterization (Ding et al., 2019) principle. Hence, we built our lightweight encoder following the structure of RepViT to improve the efficiency of echocardiogram view classification. We built the lightweight student encoder and lightweight teacher encoder for our SPEMix. To satisfy our view classification tasks, our RepViT lightweight encoder only had four blocks.

Inspired by the success of AutoMix (Liu et al., 2022), we also adopted the momentum update pipeline to decouple the process of augmentation of unlabeled images and the process of assigning the superclass pseudo-label. The architecture was shown in Figure 2. We constructed two lightweight encoders with identical initialized parameters, which can help SPEMix synchronize the two processes by employing end-to-end training and achieve better accuracy and generalization. Specifically, the DAMix Block in the teacher model mixed up the unlabeled data, and the SP Block in the student model generated superclass pseudo labels for unlabeled data. The parameters of the student model can be updated by back propagation, while the teacher parameters were updated by the exponential moving average strategy (Polyak and Juditsky, 1992) from the parameters of the student model. The total loss function of our SPEMix was the Equation 19:


(19) $${𝓁}_{\mathrm{total}}={𝓁}_{\mathrm{DAMix}}+{𝓁}_{SP}+{𝓁}_{\mathrm{op}}$$

The process of updating the teacher model can be reformulated as the Equation 20:


(20) $$\theta_{\mathrm{teacher}}=m\theta_{\mathrm{teacher}}+\left(1-m\right)\theta_{\mathrm{student}}$$

where m was the momentum coefficient and $m\epsilon (0,1],\theta_{\mathrm{student}}$ denoted the parameters of the student model, $\theta_{\mathrm{teacher}}$ denoted the parameters of teacher model.

## 3 Results and discussion

### 3.1 Implement details

#### 3.1.1 Dataset

We used the *Tufts Medical Echocardiogram Dataset 2* (TMED2) (Huang et al., 2022) to train the proposed SPEMix and the CAMUS (Leclerc et al., 2019) dataset and Unity (Howard et al., 2021) dataset to evaluate the generalization. Specifically, the TMED2 contains four types of echocardiogram views, including PLAX, PLSX, A2C, and A4C. This dataset provides 353,500 unlabeled images and 24,964 labeled data collected from different patients. For TMED2, we used the officially released training sets, test sets, and validation sets. All of the resolution of this dataset is 112 × 112 pixels. We used the RandomCrop and RamdomHorizontalFlip as basic augments for the training dataset during training. The CAMUS (Leclerc et al., 2019) dataset contains two view types including A2C, and A4C. We resized the resolution to 112 × 112 pixels to evaluate the generalization of the classification. The Unity (Howard et al., 2021) dataset contains three view types, including PLAX, A2C, and A4C. We resized the resolution to 112 × 112 pixels to evaluate the generalization of the classification.

#### 3.1.2 Training setting

For training our SPEMix, we used our designed lightweight encoder with 112 × 112 size inputs. For the hyper-parameter of the SPEMix, the momentum coefficient was set to 0.999. For labeled data batch size and unlabeled data batch size, we set them to 64. We used the Adam optimizer to update the model parameters. For learning rate, we chose from the set of {0.1,0.01,0.001,0.0001}, and we reported the different best learning rate in different experiments. The learning rate of the SPEMix was set to 0.0001, we trained the SPEMix 500 epochs and adapted the learning rate by the Cosine Schedule (Loshchilov and Hutter, 2022). We did not use the warm-up strategy. During the process of training, the parameters of the student model were updated via back propagation. And the parameters of the DAMix Block can update via the loss function of DAMix. The parameters of the DAMix Block will be frozen when the parameters of the teacher model update based on the parameters of student model. For comparison experiments, we used the Adam optimizers to train the other methods and we chose the best learning rate for each method. All of the comparison methods were trained on the TMED2. To make a fair comparison, all of our experiments were implemented on the GPU of the model NVIDIA A40.

### 3.2 Results of SPEMix

**Figure 6:**
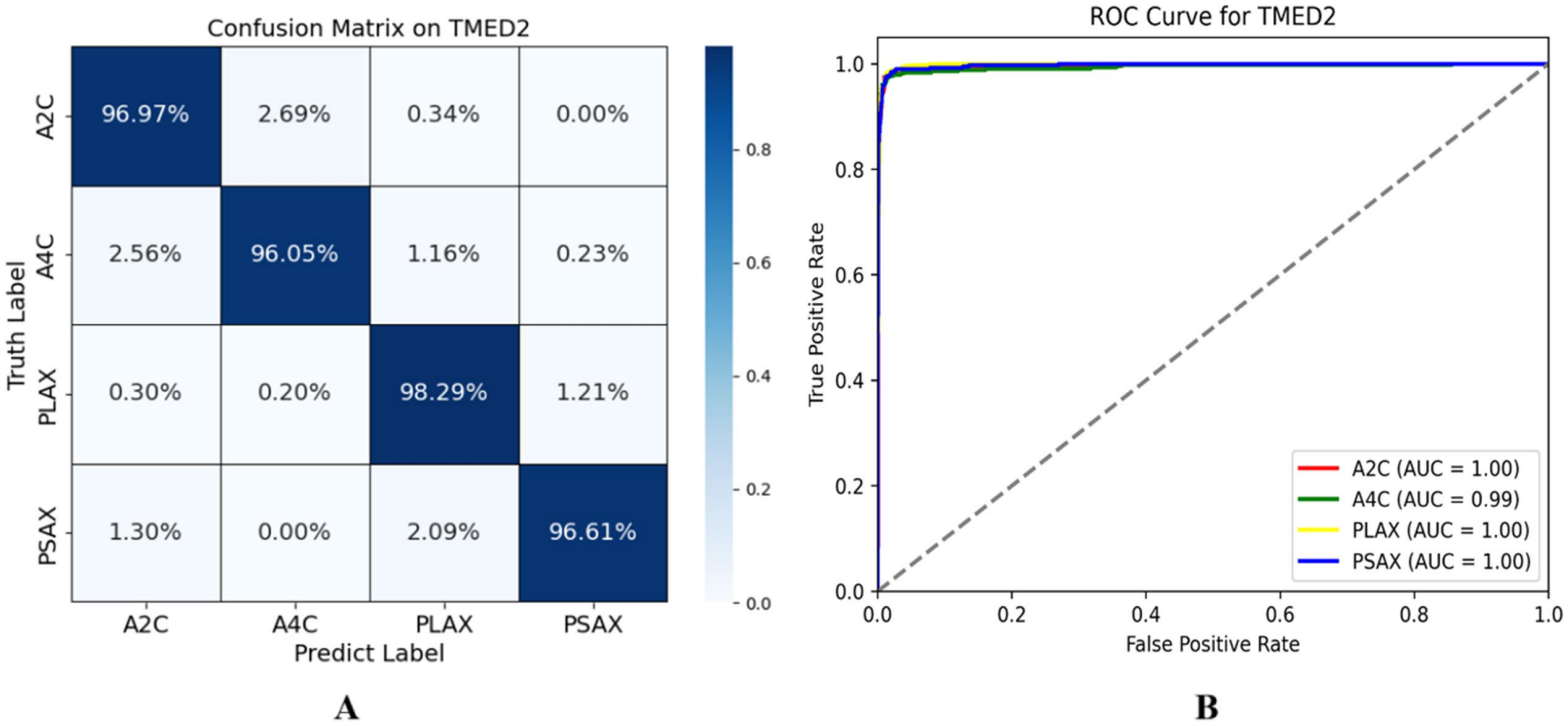
The evaluation result of the SPEMix. The left diagram has some problems due to decimal retention, so we provide a new confusion matrix on TMED2 **(A)** represents the confusion matrix of SPEMix on TMED2 and the right diagram **(B)** represents the ROC curve of TMED2.

We reported the performance of our lightweight encoder via SPEMix on the TMED2 test dataset, the confusion matrix of the test dataset was shown in the left diagram of Figure 6. Each element of the matrix represented the probability of being predicted as the corresponding class, and the diagonal value represented the prediction accuracy of each view. The classification accuracy of the A2C view reached 96.97%, the classification accuracy of A4C reached 96.05%, the PLAX classification accuracy reached 98.29%, and the PSAX classification reached 96.61%. These results demonstrated that our SPEMix predicted every view very well. At the same time, we also provided the ROC curve to evaluate our classifier. We gave the ROC curve of SPEMix and calculated the AUC for each class. The larger values of the AUC mean the better the performance of our classifier.

### 3.3 Comparison results with mixing methods

In this section, we compared our proposed SPEMix with the previous SOTA mixup methods to prove the advanced performance of our SPEMix, including CutMix (Yun et al., 2019), SaliencyMix (Uddin et al., 2006), and AutoMix (Liu et al., 2022). CutMix represents the typical mixup method in natural images. SaliencyMix is the SOTA mixup that can generate mixed data by utilizing the saliency information of the natural images. AutoMix is the SOTA of the pixel-level mixup method which gets the most advanced performance in natural images. To compare these methods fairly, we, respectively, trained a common WideResNet encoder classifier and our lightweight encoder classifier by using each mixup method. We chose the best parameters for all of the methods in this comparison experiments. The best learning rate was set to 0.01. The batch size was set to 64, we all used the Cosine Schedule to adapt the learning rate and the training epoch was set to 500. All of the experiments did not use the warm-up strategy. The comparison result was shown in Table 1. We reported the mean accuracy and standard deviation on the TMED2 from three different trails.

**Table 1:** Comparison results between SPEMix and the previous Sota mixing methods. All results are expressed as percentages (%).

Method	Encoder	Test accuracy
CutMix (Yun et al., 2019)	Wideresnet (Zagoruyko and Komodakis, 2016)	94.30 ± 0.2
CutMix (Yun et al., 2019)	Lightweight encoder(Ours)	95.97 ± 0.67
SaliencyMix (Uddin et al., 2006)	Wideresnet (Zagoruyko and Komodakis, 2016)	96.31 ± 0.43
SaliencyMix (Uddin et al., 2006)	Lightweight encoder(Ours)	96.87 ± 0.2
AutoMix (Liu et al., 2022)	Wideresnet (Zagoruyko and Komodakis, 2016)	96.34 ± 0.19
AutoMix (Liu et al., 2022)	Lightweight encoder(Ours)	96.91 ± 0.27
SPEMix(Ours)	Wideresnet (Zagoruyko and Komodakis, 2016)	**97.21 ± 0.10**
SPEMix(Ours)	Lightweight encoder(Ours)	**97.28 ± 0.11**

To make a fair comparison, we ran each method with three trials and reported the mean and standard deviation of test accuracy on the TMED2 dataset. The best performance of each encoder was highlighted in bold.

**Figure 7:**
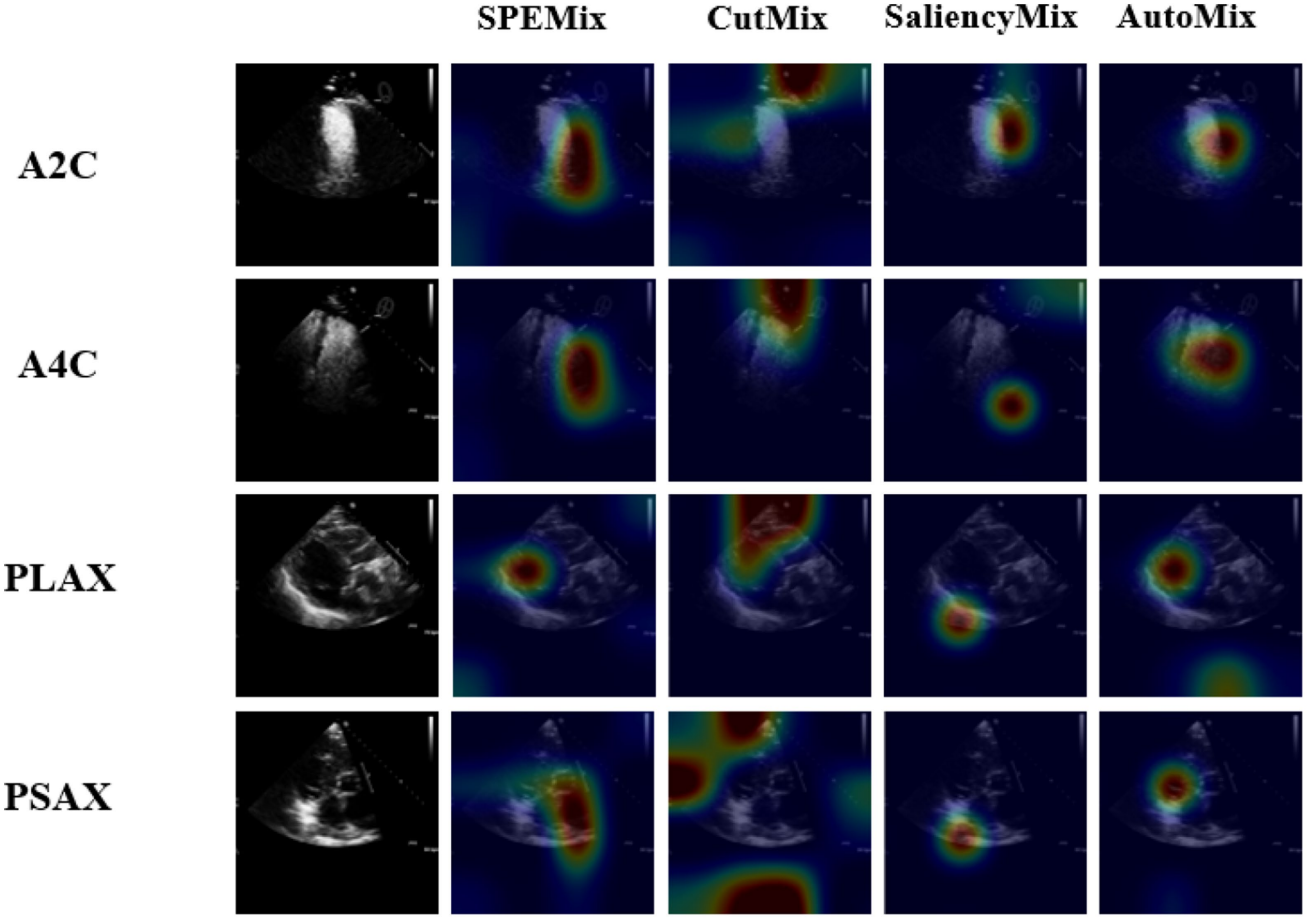
The class activation mapping (CAM) for classifiers that are trained based on different mixing methods. The data of each view is chosen from the validation datasets randomly. The visualization results of regions focused on lightweight models trained by different methods. Our proposed SPEMix can better focus on the vital regions than other SOTA methods.

To better investigate the classification process of each method, we visualized the performance of each method via the technique of CAM (Selvaraju et al., 2017). The visual results of the comparison experiment are shown in Figure 7. From the visual result, we can intuitively observe that the proposed SPEMix is more adept at focusing on crucial information compared to other advanced data augmentation methods. Based on the comparison experiment results in Table 1 and Figure 7, we got the following conclusions:

1. Our proposed SPEMix is less affected by the echocardiogram’s background information than CutMix. Our SPEMix can improve by 2.91% with the encoder of Wideresnet and improve by 1.31% with the lightweight encoder compared to CutMix. To better understand the experimental results, we compared the visualization results of CutMix and SPEMix in Figure 7. These results illustrated that CutMix focuses more on the background information of the echocardiogram. This is mainly because cutmix performs mixup augmentation by randomly selecting regions of the image. This approach results in the appearance of augmentation data containing only background information, leading to the trained model not differentiating well between the foreground and background regions of a medical image. Hence, the classification performances of Cutmix are more affected by irrelevant information.
2. Our proposed SPEMix focused on more regions containing salient echocardiogram information than SaliencyMix. Our SPEMix improved by about 0.9% with the encoder of Wideresnet and about 0.41% with the encoder of RepViT compared to SaliencyMix. The saliency information helped our SPEMix and SaliencyMix focus on the vital information from the visual results in Figure 7. However, the SPEMix focused more regions on the salient information of the echocardiographic foreground region when performing view classification. The main reason is that our SPEMix can include more salient details in the foreground by seeking detailed salient information at the pixel level. However, SaliencyMix seeks salient areas at the image level, and this method cannot consider the detailed features.
3. Our proposed SPEMix sought vital information more easily from an echocardiogram than Automix. Our SPEMix can improve by 0.87% with the encoder of Wideresnet and by 0.37% with the encoder RepViT compared to AutoMix. These visualization results illustrated that SPEMix more easily focused on vital information. These results were mainly because our SPEMix generated the mixed mask with the assistance of dynamic attention. The mixed masks containing vital information helped the model easily seek important details. However, Automix generated the mixed mask only considering the similarity of the two images. The background information of the medical images was also similar between different views. This background information prevented the model from seeking vital information.
4. Furthermore, the experimental results also revealed the potential of the designed lightweight network for application in different methods. The designed lightweight network improved the performance of TMED2 compared to the traditional WideResnet encoder using various techniques for training. In CutMix, SaliencyMix, and AutoMix, the lightweight network enhanced by 1.67%, 0.56%, and 0.57%, respectively. Using our SPEMix method for training, the lightweight network still maintains better performance. The experimental results demonstrate the greater feasibility of lightweight networks in view classification.

In summary, our SPEMix can achieve the best test classification accuracy on the WideResNet encoder and our lightweight encoder compared to other mixup methods. These experiment results demonstrate our SPEMix has advanced performance in view classification tasks.

### 3.4 Comparison results with semi-supervised learning

In this section, we compared the performance of SPEMix with other competitive semi-supervised learning methods, which include FixMatch (Sohn et al., 2020), Fix-a-step (Huang et al., 2023), OpenMatch (Saito et al., 2021), and InterLUDE (Huang et al., 2024). We test different methods on TMED2, CAMUS, and Unity datasets to evaluate the accuracy and generalization of these methods. We trained the FixMatch and OpenMatch with the optimal parameters. Specifically, each method was trained with our lightweight encoder, the Adam optimizer, and 500 epochs. The learning rate was set to 0.001. We also trained Fix-a-step with the optimal parameters(Wideresnet as encoder and SGD optimizer) given in their paper to ensure that their method can achieve the most accurate classification results. For the InterLUDE and IntereLUDE+(IntereLUDE with Self-Adaptive Threshold and Self-Adaptive Fairness), we directly quote the results given in the paper(the code is not yet open source). The comparison results for accuracy and training time are presented in Tables 2, 3, respectively.

**Table 2:** The performance of different semi-supervised methods on three datasets. All results are expressed as percentages (%).

Methods	TMED2 (Huang et al., 2022)	CAMUS (Leclerc et al., 2019)	Unity (Howard et al., 2021)
FixMatch (Sohn et al., 2020)	95.49 ± 0.07	81.39 ± 0.72	91.41 ± 1.28
$\mathrm{Fix}-a-step^{†}$ (Huang et al., 2023)	95.64 ± 0.16	83.20 ± 0.74	93.72 ± 0.65
OpenMatch (Saito et al., 2021)	96.31 ± 0.06	83.37 ± 2.42	92.11 ± 1.08
IntereLUDE* (Huang et al., 2024)	96.55 ± 0.39	86.25 ± 6.22	**96.14 ± 0.48**
IntereLUDE+* (Huang et al., 2024)	96.75 ± 0.17	81.88 ± 8.37	94.47 ± 0.85
SPEMix(Ours)	**97.28 ± 0.11**	**87.64 ± 0.67**	94.71 ± 0.24

^†^Means the method follows the parameters of the cited work with the encoder of Wideresnet and uses SGD optimizer. *Means the results cited from the SOTA work. Other methods used our lightweight encoder and the Adam optimizer. The best performances of different datasets in bold.

**Table 3:** The comparison results of training time between the traditional semi-supervised method and SPEMix.

Method	Total time	Average time
OpenMatch	4595.57 s	15.32 s
FixMatch	2746.83 s	9.16 s
SPEMix(Ours)	2714.18 s	9.05 s

From the comparison results, we found that our SPEMix can achieve better accuracy on the three datasets. Compared to the FixMatch, the accuracy, and generalization of our SPEMix are improved by 1.79%, 6.25%, and 3.3% on TMED2, CAMUS, and Unity, respectively. The reason is that the proposed SP Block can leverage the out-of-distribution data to improve the accuracy. FixMatch focuses on the close-set problem and does not pay attention to the out-of-distribution echocardiograms so FixMatch has worse results and is not suitable for open-set tasks (Saito et al., 2021). Meanwhile compared to OpenMatch, the accuracy of our SPEMix is improved by 0.97%, 4.27%, and 2.6% on TMED2, CAMUS, and Unity, respectively. The reason is that our SPEMix can also gain more improvement from the unlabeled datasets via the proposed mixed unlabeled consistency regularization. However, OpenMatch filtered out out-of-distribution data during the training process via its consistency regularization to process the open set data. In this way, OpenMatch neglected some vital information of out-of-distribution so that it has worse results. Compared to the advanced method for similar medical tasks, Fix-a-step, the SPEMix also improves the classification accuracy on different three datasets. Specifically, the accuracy and generalization of SPEMix compared to Fix-a-step improved by 1.64%, 4.44%, and 0.99% on TMED2, CAMUS and Unity, respectively. The reason for these results is that our SPEMix modeled the out-of-distribution data using superclass distribution to utilize all of the unlabeled data efficiently. However, Fix-a-step only used limited unlabeled data, which can improve the classification accuracy. In this way, Fix-a-step only got finite information from the limited out-of-distribution data. Simultaneously, our SPEMix also compares recent SOTA semi-supervised methods in view classification tasks, IntereLUDE and IntereLUDE+(IntereLUDE with Self-Adaptive Threshold and Self-Adaptive Fairness). Compared to the IntereLUDE, our SPEMix can improve by 0.73% and 1.39% on TMED2 and CAMUS. Compared to the IntereLUDE+, our SPEMix can improve by 0.53%, 5.76%, and 0.24% on TMED2, CAMUS, and Unity, respectively. SPEMix has less accuracy in the Unity dataset than IntereLUDE. However, IntereLUDE evaluated the accuracy on the widereset and did not explore a lightweight model. Therefore, our SPEMix still outperforms IntereLUDE overall.

These demonstrate that the accuracy and generalization of our proposed SPEMix outperform the SOTA methods in the view classification task. In summary, all of these results indicate the superior performance and generalization ability of the proposed SPEMix.

### 3.5 Comparison results between different encoders

In this section, we explored the performance of our proposed lightweight encoder. We reported the number of parameters of the different encoders used in our comparison experiments of Section 3.3, i.e., the number of the parameters of the WideResNet-28-2 and our proposed lightweight encoder. We also reported the accuracy of the two encoders on the TMED2 test dataset after training via SPEMix. The comparison results were shown in Table 4.

**Table 4:** The comparison results of different encoders in our comparison experiments. All accuracies are expressed as percentages (%).

Encoder model	Parameters	Accuracy
WideResNet-28-2 (Zagoruyko and Komodakis, 2016)	5.93 M	97.24
Lightweight encoder(Ours)	0.70 M	97.34

The results in Table 4 demonstrated that the parameter number of our proposed encoder is reduced by 88.2% compared with the parameter number of the WideResNet-28-2. Additionally, the results also demonstrated that our proposed lightweight encoder can also maintain the classification performance compared with the WideResNet-28-2 when training via the proposed SPEMix. Otherwise, these results of Table 1 also denoted that, with different mixing methods, our proposed lightweight encoder was better than the WideResNet-28-2. These results demonstrated that our proposed SPEMix got better performance with fewer parameters. Hence, our proposed method had the potential for clinical application.

### 3.6 Results of ablation experiments

The proposed SPEMix included two core components, DAMix and SP Block, to perform the mixed data augmentation and superclass pseudo-label generation, respectively. In order to explore the effect of each component in SPEMix, the lightweight network was regarded as the baseline in the ablation experiment, and we added DAMix Block and SP Block to the baseline step by step. The final results of the ablation experiments were in Table 5.

**Table 5:** The results of the ablation experiment. All results are expressed as percentages (%).

	TMED2 (Huang et al., 2022)	CAMUS (Leclerc et al., 2019)	Unity (Howard et al., 2021)
Baseline	94.2	74.93	85.07
Baseline+DAMix	96.02	81.56	91.11
Baseline+SP Block	96.21	82.56	93.36
SPEMix	97.34	87.11	94.42

The baseline was the proposed lightweight encoder. We trained the baseline by using the TMED2 and reported the classification accuracy on different test datasets.

Through the results of the ablation experiment, we can observe that the DAMix Block improved the baseline by 1.82%, 6.63%, and 6.04% on TMED2, CAMUS, and Unity. These results demonstrated the Efficient Mixup based on our proposed DAMix Block can improve accuracy. Furthermore, the SP Block improved the baseline by 2.01%, 0.73%, and 8.29% on TMED2, CAMUS, and Unity. The results denoted the advancement of utilizing the out-of-distribution data from the superclass probability perspective. The SPEMix can increase the baseline by 3.14%, 12.18%, and 9.35% on TMED2, CAMUS, and Unity, which illustrates the SPEMix can fuse the advantages of the DAMix Block and SP Block. These results demonstrated the effectiveness of the proposed SPEMix. The DAMix Block can generate corresponding mixed masks embedded with a mixing ratio for unlabelled data. The high-quality mixed data generated through the mixed masks can improve classification accuracy and generalization. SP Block can assign superclass pseudo-labels to unlabelled data through the perspective of superclass distribution to make use of the out-of-distribution data. This out-of-distribution information improved the classification accuracy and generilization. SP Block used an end-to-end approach to fuse two phases. Firstly, the unlabelled mixed data was generated using DAMix, while the information of generated mixed data was leveraged by assigning superclass pseudo-labels through SP Block. The high-quality mixed data generated by DAMix can also enrich the information of out-of-distribution that be leveraged by the SP Block.

## 4 Conclusion

In this work, we proposed a novel lightweight open-set semi-supervised learning method, SPEMix, to improve the accuracy and generalization of the echocardiogram view classification. The proposed DAMix Block generated the masks embedded with the mixing ratio containing vital information. These masks efficiently generated high-quality mixed data at the pixel level. Then, the proposed SP Block can generate the superclass pseudo-label from the superclass probability perspective to utilize the vital information from the unlabeled medical dataset. In this way, the proposed SP Block used the out-of-distribution data more effectively. Meanwhile, a novel loss function based on unlabeled consistent regularization was proposed to make the classification model better optimized from the supervision of the mixed unlabeled data and the super pseudo-label. Otherwise, we built a lightweight encoder based on RepViT to decrease the model parameters and improve the classification efficiency. Experiment results indicated that our proposed SPEMix achieved better performance and generalization than other semi-supervised learning methods. Our SPEMix had the potential for clinical application. Although the proposed SPEMix method shows encouraging results for echocardiogram view classification, there are still several areas that require further investigation. Future research could aim to adapt the method for more complex or multi-modal medical datasets, incorporating additional imaging modalities (such as CT or MRI) and patient metadata into the semi-supervised framework.

## Data Availability

The Tufts Medical Echocardiogram Dataset 2 (TMED2) (Huang et al., 2022) was used to train the proposed SPEMix. The CAMUS (Leclerc et al., 2019) dataset and Unity (Howard et al., 2021) dataset were used to evaluate the generalization.
